# Assessment of genomic prediction accuracy using different selection and evaluation approaches in a simulated Korean beef cattle population

**DOI:** 10.5713/ajas.20.0217

**Published:** 2020-06-24

**Authors:** Chiemela Peter Nwogwugwu, Yeongkuk Kim, Hyunji Choi, Jun Heon Lee, Seung-Hwan Lee

**Affiliations:** 1 Division of Animal and Dairy Science, Chungnam National University, Daejeon 34134, Korea

**Keywords:** Heritability, Marker Density, Prediction Accuracy, Simulation, Selection Method

## Abstract

**Objective:**

This study assessed genomic prediction accuracies based on different selection methods, evaluation procedures, training population (TP) sizes, heritability (h^2^) levels, marker densities and pedigree error (PE) rates in a simulated Korean beef cattle population.

**Methods:**

A simulation was performed using two different selection methods, phenotypic and estimated breeding value (EBV), with an h^2^ of 0.1, 0.3, or 0.5 and marker densities of 10, 50, or 777K. A total of 275 males and 2,475 females were randomly selected from the last generation to simulate ten recent generations. The simulation of the PE dataset was modified using only the EBV method of selection with a marker density of 50K and a heritability of 0.3. The proportions of errors substituted were 10%, 20%, 30%, and 40%, respectively. Genetic evaluations were performed using genomic best linear unbiased prediction (GBLUP) and single-step GBLUP (ssGBLUP) with different weighted values. The accuracies of the predictions were determined.

**Results:**

Compared with phenotypic selection, the results revealed that the prediction accuracies obtained using GBLUP and ssGBLUP increased across heritability levels and TP sizes during EBV selection. However, an increase in the marker density did not yield higher accuracy in either method except when the h^2^ was 0.3 under the EBV selection method. Based on EBV selection with a heritability of 0.1 and a marker density of 10K, GBLUP and ssGBLUP_0.95 prediction accuracy was higher than that obtained by phenotypic selection. The prediction accuracies from ssGBLUP_0.95 outperformed those from the GBLUP method across all scenarios. When errors were introduced into the pedigree dataset, the prediction accuracies were only minimally influenced across all scenarios.

**Conclusion:**

Our study suggests that the use of ssGBLUP_0.95, EBV selection, and low marker density could help improve genetic gains in beef cattle.

## INTRODUCTION

Genomic estimated breeding values (GEBVs) of selected individuals are frequently used to improve the genetics of economically important traits in livestock species. These values incorporate the results of evaluations using genomic data, pedigree records and the phenotypic performance of individuals using the best linear unbiased prediction (BLUP) or genomic method [1,2]. The accuracy of estimated breeding value (EBV) is an important factor that could influence the selection accuracy of breeding animals [3]. Alternatively, accuracy based on genomic selection (GS) could increase the predictive ability of the GEBV by applying single nucleotide polymorphism (SNP) information. Previous studies have reported improved GEBV accuracy, genetic gain, selection accuracy and a reduction in the generation interval for economic traits [2,4,5]. Calus [6] reported that the efficiency of GS in livestock depends on the prediction accuracy of GEBVs. However, the prediction accuracy of GEBVs can be influenced by several factors, such as methods of prediction [7], the training population (TP) size [6], the h2 of the trait [8] and the marker density [9]. An effect of errors in pedigree on the accuracy of the EBV has been reported in Korean Hanwoo cattle [10], but the use of genomic information could improve the accuracy of GEBVs.

Several authors have reported genomic prediction evaluation methods that could increase the accuracy of GEBVs. Hayes et al [2] and VanRaden [11] suggested the use of GBLUP, which employs genomic information in the form of a genomic relationship matrix. It also describes additive genetic covariance between individuals. GBLUP calculates direct genomic values (DGV) for genotyped individuals and has advantages over BLUP because marker density captures the Mendelian sampling across the genome. GBLUP is a straightforward procedure with low computational requirements, and has been used for genomic evaluations in cattle [12]. In contrast, single-step GBLUP (ssGBLUP) predicts how non-genotyped individuals can benefit from genomic information. The pedigree record and marker (SNPs) relationship matrices are combined in ssGBLUP, permitting the blending of genotyped and non-genotyped individuals in the genetic evaluation [13]. Weight (*w*) has been added to the genomic relationship matrix (G) [11], and such an adjustment may be interpreted as relative weight on the polygenic effect [14]. To facilitate inversion, w values between 0.90 and 1.0 indicate variations in prediction accuracy [11], but insignificant differences in EBVs have been reported when w ranges between 0.95 and 0.98 [14]. Recent studies have reported improved accuracies of GEBVs obtained using ssGBLUP compared with those from GBLUP in simulated beef cattle [15].

Korean Hanwoo cattle possess good meat flavour, tenderness and taste, and efforts have been made to improve the quantity and quality of the carcass [16]. Applying GS is a potential approach to improve the genetic gains in economically important traits. However, Onogi et al [17] pointed out that the use of ssGBLUP for genomic evaluations is still emerging in beef cattle due to the greater complexity of their records compared with those of other livestock species, such as the existence of pedigree errors (PEs) and fewer full or half-sib families [10,18]. On the other hand, a simulation study allows the testing of several theories, permitting an unravelling of the complex evolutionary patterns that are otherwise difficult to comprehend. For example, the history of human migration provides significant insight into the present patterns of DNA variation in humans [19]. Simulation studies in beef cattle and other livestock have provided information on their potential for genomic evaluation. They have also been used in studies of predictions of total genetic value [8], genomic prediction of simulated multi-breed and purebred cattle [20], GS accuracy in simulated populations [21] and a comparison between single- and two-step GBLUP methods in simulated beef cattle [15]. These authors reported that GS increases the accuracy of the selection and economic benefits of the breeding objective during beef production. Therefore, the present study assessed the prediction accuracy of GEBV based on different selection methods, evaluation procedures, TP sizes, h^2^ levels, marker densities and PE rates using a simulated Korean beef cattle population.

## MATERIALS AND METHODS

### Simulation

Phenotypic and genotypic data were simulated using QMSim software [22] to mimic the actual structure and extent of linkage disequilibrium (LD) that exist in beef cattle [21]. Table 1 summarises the population structure and parameters of the simulation process. Marker densities of 10K, 50K, and 777K were simulated to generate bi-allelic markers distributed across 29 autosomal chromosomes of equal length. First, a historical population (HP) was simulated, which was comprised of constant size of 1,000 individuals across 1,000 generations. The size was gradually reduced to 200 individuals (100 males and 100 females) in the subsequent 95 generations to create initial LD and mutation-drift equilibrium. In addition, the HP was based on random mating. Next, 200 individuals (effective population size, Ne) were randomly selected from the last historical generation to expand the population size. All males and females were randomly mated and each dam produced five offspring per generation for 20 generations. Finally, 275 males and 2,475 females were randomly chosen from the expanded population. These selected individuals were simulated across ten recent generations with one offspring per dam. Parameters that mimic Korean beef cattle were applied in the simulation based on the recent generations. Selection designs were based on the phenotypic performance and BLUP (EBV) approaches. The replacement ratios were 60% (sires) and 20% (dams). The rate of missing sire and dam records was 0.05. Traits with heritability levels of 0.1, 0.3, and 0.5 with a phenotypic variance of 1 were used. The Henderson mixed linear model was used to predict the EBVs, and the TP sizes were 1,000, 2,000, 3,000, and 5,000 individuals randomly selected from generations 7, 8, and 9 [1]. A total of 200 individuals were randomly selected from the tenth generation for the prediction set. The scenarios considered incorporated various selection methods, evaluation procedures, TP sizes, h^2^ levels and marker densities.

#### Genome

The genome was comprised of 29 pairs of chromosomes with length identical to the actual bovine genome size (2,349 cM). Marker densities of 10K, 50K, and 777K were selected such that they would produce three different densities of segregating bi-allelic loci. The effect of the markers on the traits was neutral. The whole genome consisted of 725 quantitative trait loci (QTLs), and the segregating QTLs were comprised of two, three or four alleles randomly distributed with mean allelic frequency >0.01. The additive genetic effects of the QTLs were sampled from a gamma distribution with a parametric shape equal to 0.4. The rate of missing marker genotypes was 0.01, and the rate of marker genotyping error was 0.005. To establish mutation-drift equilibrium, a recurrent mutation rate of 10^−5^ was used for the markers and the QTLs throughout the simulation. The phenotypes were produced by adding random residuals to the QTL effects.

### Simulation of pedigree errors

The simulation was performed based on the EBV selection method with a 50K marker density and heritability of 0.3, as previously explained above. However, the protocol was modified by introducing errors into the simulated pedigree dataset by randomly assigning sires to all progenies from generations 1 to 10, respectively. This method changed the sires’ information and resulted in wrong sire records. Thus, the impact of PEs was assessed in generations 7, 8, and 9 with their averages. The SampleBy function in the doBy package of the R software package (The R Foundation for Statistical Computing, Vienna, Austria) was used for creating PEs. Several error rates were substituted in the pedigree dataset for each generation, such as 10%, 20%, 30%, and 40%, respectively, as described previously by Oliehoek and Bijma [23] and adopted by Nwogwugwu et al [10].

### Methods for genomic prediction

#### GBLUP procedure

The genotyped individuals were used to estimate the DGVs using the following model:

y=1μ+Zg+e,

where *y*, *μ*, *g*, and *e* are the vectors of the phenotypes, overall mean, DGV and residual errors, respectively, and *Z* is the incidence matrix that relates phenotypes to random marker effects (DGVs). $\text{g}\sim N\;(0,\;G\sigma_{g}^{2})$, where $\sigma_{g}^{2}$ is the additive genetic variance and g is the marker-based genomic relationship matrix [11]. Random residuals were assumed such that $\text{e}\sim N\;(0,\;\text{I}\sigma_{e}^{2})$, where $\sigma_{e}^{2}$ is the residual variance. The G matrix was calculated using the SNP genotype data as follows:

$$\text{G}=\frac{(\text{M}-\text{P}){\left(\text{M}-\text{P}\right)}^{\prime }}{2\sum_{j=1}^{m}Pj(1-{\text{P}}_{\text{j}})}$$

where M is the marker allele matrix for each individual and P is a matrix comprising the frequency of the second allele (p*_j_*), noted as 2(p*_j_*). GS3 software was used to perform the GBLUP analyses [24].

#### ssGBLUP procedure

This procedure employed data from genotyped and non-genotyped individuals and used the inverse of the H matrix (H^−1^) to blend the pedigree-based matrix (A) with the genomic relationship matrix (G) as described previously [25].

$$H^{-1}=A^{-1}+\left(\begin{array}{cc} 0 & 0\\ 0 & {\left(wG+(1-w)A_{22}\right)}^{-1}-A_{22}^{-1} \end{array}\right)$$

where *w* is a constant for the weighting factor as described by Abdalla et al [26], $A_{22}^{-1}$ is the inverse A matrix for genotyped individuals and G is explained above. Three different weights (0.95, 0.90, and 0.85) were suggested to prevent possible problems with inversion and to explain the relative weight of the polygenic effect required to describe the total additive variance. These different weight values are indicated as ssGBLUP_0.95, ssSGBLUP_0.90, and ssGBLUP_0.85, respectively. For more information, see [26]. The BLUPF90 software was used for the ssGBLUP predictions [27].

### Accuracy of the prediction procedure

To evaluate the ability of genomic prediction for each set of marker densities, TP sizes of 1,000, 2,000, 3,000, and 5,000 individuals were randomly chosen from generations 7 to 9. In total, 200 individuals were randomly selected from generation 10 for the prediction set. The accuracies of the predictions are usually accessible from genetic evaluations and can be determined from the additive genetic variance and the prediction error variance (PEV), as described previously [28]. The accuracy of GEBV was calculated as:

$$\sqrt{\frac{1-\text{PEV}}{\sigma_{\text{g}}^{2}}}$$

where PEV is the prediction error variance of EBV, and $\sigma_{\text{g}}^{2}$ is the additive genetic variance of each trait.

## RESULTS

### Accuracy of genomic predictions based on phenotypic selection across all scenarios

Based on the phenotypic method of selection, the prediction accuracies for genomic evaluation were estimated under different scenarios (e.g., 10K, 50K, and 777K; h^2^ = 0.1, 0.3, and 0.5; different TP sizes), as shown in Table 2. The GBLUP prediction accuracies ranged from 0.312 to 0.563 for an h^2^ of 0.1, 0.475 to 0.735 for an h^2^ of 0.3 and 0.575 to 0.808 for an h^2^ of 0.5, respectively. For ssGBLUP_0.95, the accuracies of prediction ranged from 0.341 to 0.566, 0.519 to 0.740, and 0.630 to 0.813 for heritabilities of 0.1, 0.3, and 0.5, respectively. These results further indicate that the prediction accuracy of ssGBLUP _0.95 increased by 8.77%, 4.30%, 2.06%, and 0.50% across TP sizes of 1,000, 2,000, 3,000, and 5,000 with a heritability of 0.1 and a marker density of 10K when compared with GBLUP. The prediction accuracy of GBLUP and ssGBLUP_0.95 dramatically increased with an increase in TP size. The lowest accuracy was obtained for a TP size of 1,000, whereas the highest occurred with a TP size of 5,000 individuals. An increase in the heritability level also improved the accuracy of prediction. The lowest prediction accuracy was found for an h^2^ of 0.1, whereas the highest was observed when the h^2^ was increased to 0.5. In contrast, the prediction accuracy of the genomic methods did not improve with an increase in the marker density. A 10K marker density showed better prediction accuracy, followed by those of 50K and 777K. More precisely, the highest accuracy of genomic predictions was observed when the marker density was 10K at an h^2^ of 0.5 and a TP size of 5,000.

### Accuracy of genomic predictions based on EBV selection across all scenarios

Tables 3, 4, and 5 summarise the EBV method of selection and prediction accuracies for GBLUP and ssGBLUP with three different combinations of weights (*w*) when the marker density ranged from 10K to 777K; h^2^ = 0.1, 0.3, or 0.5; and the TP size was varied between 1,000 and 5,000. The prediction accuracies for the GBLUP and ssGBLUP approaches using the EBV selection method were higher than those using phenotypic selection. The prediction accuracies for GBLUP ranged from 0.367 to 0.605 for an h^2^ of 0.1, 0.496 to 0.754 for an h^2^ of 0.3 and 0.608 to 0.822 for an h^2^ of 0.5. ssGBLUP 0.95 had an accuracy of 0.397 to 0.606 for an h^2^ of 0.1, 0.545 to 0.758 for an h^2^ of 0.3 and 0.657 to 0.827 for an h^2^ of 0.5. The prediction accuracy was highest with ssGBLUP_0.95. These results indicate that the prediction accuracies obtained using ssGBLUP_0.95 increased by 9.48%, 4.25%, 2.07%, and 0.16% across TP sizes of 1,000, 2,000, 3,000 and 5,000 with a heritability of 0.1 and a marker density of 10K compared with those obtained using GBLUP. This study further revealed that the differences in prediction accuracy between ssGBLUP_0.95 and GBLUP were not large when the TP size was increased from 3,000 to 5,000. On the other hand, GBLUP increased by 15.67%, 11.63%, 9.71%, and 7.46% for TP sizes of 1,000, 2,000, 3,000, and 5,000 compared with phenotypic selection. The trend was similar for SSGLUP_0.95, but the increases were 16.2%, 11.87%, 9.71%, and 7.06%, respectively. The findings also show that increasing the number of genotyped animals in the TP sizes increased the prediction accuracies of GBLUP and ssGBLUP_0.95. Different levels of h^2^ significantly influenced the prediction accuracy across all scenarios. Increases in heritability also increased the prediction accuracy of the genomic evaluations. For example, changing the h^2^ from 0.1 to 0.5 increased the accuracy of GBLUP and ssGBLUP_0.95 by 84.95% and 84.14%, respectively, for a TP size of 1,000. The effect of marker density on the prediction accuracy of the GBLUP and ssGBLUP_0.95 methods followed a trend similar to that observed for phenotypic selection. However, with an h^2^ of 0.3 across all TP sizes, the prediction accuracies for both evaluation methods slightly increased from the 10K to 50K marker density but declined at 777K. The highest accuracy of genomic predictions was observed when the marker density was 10K, the h^2^ was 0.5 and the TP size was 5,000.

### Accuracy of genomic predictions based on EBV selection under pedigree errors

The accuracy of predictions was investigated by introducing different proportions of error into a simulated pedigree dataset while using a marker density of 50K, a heritability of 0.3, and three different weighted values of ssGBLUP (w = 0.95, 0.90, and 0.85) across the various TP sizes (Table 6). Introducing errors into the pedigree dataset slightly affected the prediction accuracies across three different weights and TP sizes. The prediction accuracy was 0.6162 with no PE based on a TP size of 1,000 with ssGBLUP_0.95, but the accuracies slightly declined to 0.6132, 0.6118, 0.6093, and 0.6075 when errors were introduced at 10%, 20%, 30%, and 40%, respectively. With a 10% PE, the decline was 0.003. The results further revealed that the accuracy decreased to 0.0087 at 40% PE. The presence of PE somewhat affected the accuracy of prediction across TP sizes. However, as the TP size increased, the effect of PE on the prediction accuracy was insignificant.

## DISCUSSION

In our study, we investigated the prediction accuracy of GEBV under different selection scenarios, evaluation procedures, TP sizes, heritability levels, marker densities and PE rates in a simulated Korean beef cattle population. Phenotypes are frequently used for selecting superior individuals in a population. However, the statistical method of evaluation used is one factors that could influence the prediction accuracy of GEBV (Table 2). With phenotypic selection, there was a higher prediction accuracy for ssGBLUP_0.95 than for GBLUP, indicating that ssGBLUP_0.95 had advantages over GBLUP, possibly due to the combination of both genotyped and non-genotyped individuals. The combination could also help genomic markers capture any QTL effect or polygenic effect through EBVs [2,4]. Our results agree with those of Gowane et al [29], who obtained a higher prediction accuracy using ssGBLUP than GBLUP in a simulated population.

The accuracy of genomic prediction improved with greater numbers of individuals, as ssGBLUP showed a higher prediction accuracy than GBLUP at TP sizes of 1,000 to 3,000. However, when the TP size was 5,000, the results of both methods were comparable. The results further revealed that increasing the TP size across different scenarios improved the prediction accuracies. Several authors have reported improved accuracy for GEBVs when increasing the TP size in genotyped Holstein bulls [4] and simulated beef cattle [21].

The accuracy of the genomic predictions was affected by increased heritability. ssGBLUP showed a higher accuracy than GBLUP at all h^2^ levels. Nwogwugwu et al [10] stated that the higher the h^2^, the better the accuracy because h^2^ represents the strength of the association between the phenotype and breeding values. This implies that there is an association between h^2^ and accuracy, as we observed; Kolbehdari et al [30] reported similar results. Numerous studies have demonstrated increased accuracy with increasing h^2^ values, which agrees with our study [21,29].

The impact of marker density on the accuracy of genomic predictions has been examined in previous study [9]. With increases in marker density of 50K and 777K, the accuracies of the genomic evaluations did not improve. Zhu et al [31] reported limited prediction accuracy of a genomic evaluation with an increase in marker density from 0.5K to 20K in live weight, carcass weight and average daily gain. However, an increase in the marker density had a conflicting effect on prediction accuracy due to co-linearity between the effects of the markers in a simulated population [32]. Some authors have reported slightly improved accuracy of GEBVs with an increase in the marker density [21]. Nevertheless, these differences in results may be due to the genetic architecture or population structure.

The use of individual EBVs has greatly aided in animal genetic improvement. Therefore, selecting individuals based on the EBV could increase the accuracy of genomic predictions (Tables 3, 4, and 5). This study examined the prediction accuracies of GEBVs across multiple scenarios. The prediction accuracies of GBLUP and ssGBLUP_0.95 were higher when using EBV selection than when using phenotypic selection. This could be attributed to the impact of the pedigree relationship among individuals, which facilitates accurate sire selection decisions. Our results agree with Amari [33], who previously stated that the EBV provides the most dependable information on the breeding results for a particular animal.

The performance of the ssGBLUP_0.95 method of prediction was superior to that of GBLUP in all scenarios. Therefore, combining genomic and pedigree data to predict traits improves accuracy, which leads to improved genetic gain in beef cattle breeding. However, GBLUP has been broadly utilised for genomic assessments in dairy cattle [7]. This assumes that the GBLUP method is mainly based on the LD between markers and QTL. On the other hand, Meuwissen et al [8] proposed that an evaluation based on a combination of models improves the accuracy of prediction compared to methods that assume all SNPs have predictive value. Three different weights were added to ssGBLUP to solve the collinearity problem between variables and the low rank of the matrix, which could make inversion of the matrix difficult or impossible. ssGBLUP_0.95 had the highest prediction accuracy compared with weights 0.90 and 0.85. The prediction accuracy of ssGBLUP_0.90 was comparable with that of GBLUP in some scenarios. Less bias and a high prediction accuracy were reported by Vitezica et al [34] when the G matrix was adjusted with a weight factor using the ssGBLUP method. Similar observations have been reported in turkey [26]. The present findings further indicated significant differences in prediction accuracies among the weights used in this study and revealed that a weighting factor of 0.95 could be an optimal choice for genetic improvement. A higher accuracy of GEBV with ssGBLUP has been reported in Japanese black cattle [17], a simulated cattle population [29] and Hanwoo beef cattle [35], indicating that the ssGBLUP method could be effectively used to improve traits with low heritability as well as traits that are difficult to measure.

The present results indicate that the prediction accuracies of traits with a higher h^2^ are more precise than those for traits with a lower h^2^. This implies that the amount of additive genetic variance explained by markers is small with a low h^2^, thereby reducing the prediction accuracy [36]. The present study further investigated the effect of TP size on the prediction accuracy. The findings showed that the prediction accuracy of genomic evaluations improved as the TP size increased, suggesting that the prediction accuracy tends to increase as information from an increasing number of individuals is added. The results also indicate that the TP size is important for successful genomic prediction. Previous study has shown increased prediction accuracy with increasing TP size [4]. As shown in Tables 3, 4 and 5, that ssGBLUP_0.95 resulted in a higher accuracy at TP sizes of 1,000 to 2,000 individuals compared with GBLUP, and an even higher with a TP size of 3,000; however, both methods were comparable above a TP size of 4,000. Our results fully agree with those of VanRaden et al [4], who observed that genomic gains increase almost linearly with an increase in TP size in Holstein bulls.

The effect of marker density on prediction accuracies was similar to that found for phenotypic selection. However, with an h^2^ of 0.3, the prediction accuracies improved with an increase in marker density of 50K, whereas the accuracy of prediction declined with the 777K marker. Genomic predictions did not improve at 800K or in transcriptome panels over 50K in a pure-breed population [37]. Wang et al [38] also reported a similar result after increasing the marker density from 0.05K to 3.2K, which greatly improved the genomic prediction accuracy, but there was less improvement when the marker density increased further. The present findings reveal high accuracies with a 10K marker density and a heritability of 0.1 and a TP size of 5,000; however, a heritability of 0.5 with a TP size of 5,000 produced the highest prediction accuracies in both models. The findings of the present study differ slightly from previous studies possibly due to variations in the genetic structure, marker density, TP size and method of evaluation.

Several authors have reported the effect of PEs on the EBV, the accuracy of the EBV and the genetic gain in livestock species [10,39]. Their findings indicate that PEs greatly reduce the accuracy of the EBV in beef and dairy cattle. However, introducing genomic information may resolve this reduction in the accuracy of the EBV or GEBV in livestock breeding. The results shown in Table 6 demonstrate the accuracy of predictions under PEs using a 50K marker density, an h^2^ of 0.3, and the use of ssGBLUP with three different weight values (0.95, 0.90, and 0.85) across TP sizes. In this study, the prediction accuracy was only moderately influenced by different weights and TP sizes. The findings further reveal that the prediction accuracy decreased consistently as more PEs were introduced into the data. This suggests that PEs have a negative relationship with prediction accuracy. Nwogwugwu et al [10] reported that the accuracy of the EBV decreased by 0.02 with a 40% PE from that generated with an estimate at 0% PE; however, with a 40% PE combined with genomic information, the prediction accuracy of the GEBV declined by only 0.003 from that obtained using 0% PE and ssGBLUP_0.95. This indicates that the accuracy of prediction based on PE combined with genomic information is more reliable than the accuracy of EBV. With increasing TP size, the effect of PE on prediction accuracy was lower or negligible. This implies that additional information from relatives or increasing the TP size may improve the prediction accuracy, even if the pedigree is erroneous.

## CONCLUSION

In general, selecting individuals based on the EBV had positive effects on the prediction accuracies of GEBVs compared with phenotypic selection, suggesting that assessing the pedigree records, phenotypic performance and genomic information of individuals improves the accuracy of GEBVs. Larger differences in the prediction accuracy between the GBLUP and ssGBLUP_0.95 methods were observed for traits with low heritability. This study showed that ssGBLUP_0.95 outperformed GBLUP under all scenarios, and could be implemented for GS. Furthermore, increasing the TP size and h^2^ improved the prediction accuracies, whereas increasing the marker density did not improve accuracy of either method except in the case of a heritability of 0.3 and use of the EBV selection method. This study further revealed that PE slightly influenced the prediction accuracies using different weights in ssGBLUP. The selection methods, evaluation procedures, TP sizes, h^2^ levels, marker densities and PEs should be considered for genetic improvement and to properly implement GS in Korean Hanwoo breeding.

## Figures and Tables

**Table 1:** Population structure and simulation parameters

Parameter	value
Step 1: Historical generations (HG)	
Number of generations(size) - phase 1	1,000 (1,000)
Number of generations(size) - phase 2	95 (200)
Step 2: Expanded generations (EG)	
Number of founder males from HG	100
Number of founder females from HG	100
Number of generations	20
Number of offspring per dam	5
Step 3: Recent generations	
Number of founder males from EG	275
Number of founder females from EG	2,475
Number of generations	10
Number of offspring per dam	1
Ratio of male	50%
Mating system	Selective
Replacement ratio for males	60%
Replacement ratio for females	20%
Selection/culling	EBV/phenotype
BV estimation method	BLUP animal model
Ratio of missing sire and dam	5%
Heritability of the trait	0.1, 0.3, or 0.5
Phenotypic variance	1.0
Genome	
Number of chromosomes	29
Total length	2,349 cM
Number of markers	10K, 50K, 777K
Marker distribution	Evenly spaced
Number of QTL	725
QTL distribution	Random
MAF for markers	0.1
MAF for QTL	0.1
Additive allelic effects for markers	Neutral
Additive allelic effects for QTL	Gamma distribution (shape = 0.40)
Rate of missing marker genotypes	0.01
Rate of marker genotyping error	0.005
Rate of recurrent mutation	0.0001

BV, breeding value; QTL, quantitative trait loci; MAF, minor allele frequency.

**Table 2:** Accuracies of genomic prediction using the GBLUP or ssGBLUP_0.95 procedures with the phenotypic selection method and various levels of heritability across TP sizes and marker densities

h^2^	TP size	Marker density					

		10K		50K		777K	
		
		GBLUP	ssGBLUP_0.95	GBLUP	ssGBLUP_0.95	GBLUP	ssGBLUP_0.95
0.1	1,000	0.319	0.347	0.314	0.346	0.312	0.341
	2,000	0.421	0.438	0.415	0.434	0.413	0.430
	3,000	0.484	0.494	0.478	0.490	0.473	0.485
	5,000	0.563	0.566	0.557	0.561	0.551	0.554
0.3	1,000	0.495	0.539	0.475	0.519	0.478	0.524
	2,000	0.606	0.630	0.588	0.610	0.588	0.612
	3,000	0.667	0.681	0.648	0.662	0.649	0.662
	5,000	0.735	0.740	0.717	0.721	0.717	0.722
0.5	1,000	0.590	0.639	0.575	0.630	0.575	0.630
	2,000	0.698	0.721	0.684	0.711	0.680	0.708
	3,000	0.752	0.765	0.738	0.754	0.734	0.751
	5,000	0.808	0.813	0.796	0.802	0.793	0.799

GBLUP, genomic best linear unbiased prediction; ssGBLUP, single-step genomic best linear unbiased prediction with weight value of 0.95 (ssGBLUP_0.95: *w* = 0.95); TP, training population size.

**Table 3:** Accuracies of genomic prediction using the GBLUP or ssGBLUP procedures with three different combinations of weights (*w*) and the EBV selection method, various levels of heritability across TP sizes and a 10K marker density

h^2^	TP size	GBLUP	ssGBLUP_0.951)	ssGBLUP_0.901)	ssGBLUP_0.851)
0.1	1,000	0.369	0.404	0.389	0.374
	2,000	0.470	0.490	0.472	0.454
	3,000	0.531	0.542	0.522	0.503
	5,000	0.605	0.606	0.586	0.565
0.3	1,000	0.526	0.570	0.552	0.533
	2,000	0.633	0.656	0.635	0.614
	3,000	0.690	0.703	0.681	0.659
	5,000	0.753	0.757	0.734	0.712
0.5	1,000	0.622	0.664	0.644	0.624
	2,000	0.722	0.742	0.720	0.698
	3,000	0.770	0.782	0.759	0.737
	5,000	0.822	0.827	0.804	0.782

GBLUP, genomic best linear unbiased prediction; ssGBLUP, single-step genomic best linear unbiased prediction; EBV, estimated breeding value; TP, training population size.

1) ssGBLUP_0.95, *w* = 0.95; ssGBLUP_0.90, *w* = 0.90; ssGBLUP_0.85, *w* = 0.85.

**Table 4:** Accuracies of genomic prediction using the GBLUP or ssGBLUP procedures with three different combinations of weights (*w*) using the EBV selection method, various levels of heritability across TP sizes and a 50K marker density

h^2^	TP size	GBLUP	ssGBLUP_0.951)	ssGBLUP_0.901)	ssGBLUP_0.851)
0.1	1,000	0.360	0.401	0.387	0.372
	2,000	0.465	0.485	0.467	0.449
	3,000	0.523	0.535	0.516	0.497
	5,000	0.596	0.597	0.577	0.557
0.3	1,000	0.532	0.577	0.559	0.540
	2,000	0.638	0.660	0.640	0.619
	3,000	0.693	0.706	0.684	0.662
	5,000	0.754	0.758	0.736	0.714
0.5	1,000	0.611	0.658	0.638	0.619
	2,000	0.710	0.733	0.712	0.690
	3,000	0.760	0.773	0.751	0.729
	5,000	0.812	0.817	0.795	0.773

GBLUP, genomic best linear unbiased prediction; ssGBLUP, single-step genomic best linear unbiased prediction; EBV, estimated breeding value; TP, training population size.

1) ssGBLUP_0.95, *w* = 0.95; ssGBLUP_0.90, *w* = 0.90; ssGBLUP_0.85, *w* = 0.85.

**Table 5:** Accuracies of genomic prediction using the GBLUP or ssGBLUP procedures with three different combinations of weights (*w*) using the EBV selection method, various levels of heritability across TP sizes and a 777K marker density

h^2^	TP size	GBLUP	ssGBLUP_0.951)	ssGBLUP_0.901)	ssGBLUP_0.851)
0.1	1,000	0.367	0.397	0.382	0.367
	2,000	0.459	0.478	0.460	0.443
	3,000	0.518	0.529	0.510	0.491
	5,000	0.593	0.594	0.574	0.554
0.3	1,000	0.496	0.545	0.527	0.510
	2,000	0.603	0.629	0.609	0.589
	3,000	0.662	0.677	0.655	0.634
	5,000	0.728	0.732	0.710	0.689
0.5	1,000	0.608	0.657	0.638	0.619
	2,000	0.708	0.733	0.711	0.690
	3,000	0.759	0.773	0.751	0.729
	5,000	0.811	0.817	0.795	0.773

GBLUP, genomic best linear unbiased prediction; ssGBLUP, single-step genomic best linear unbiased prediction; EBV, estimated breeding value; TP, training population size.

1) ssGBLUP_0.95, *w* = 0.95; ssGBLUP_0.90, *w* = 0.90; ssGBLUP_0.85, *w* = 0.85.

**Table 6:** Accuracies of genomic prediction based on EBV selection with various pedigree errors using a 50K marker density, an h^2^ of 0.3, the ssGBLUP procedure and three different weight values

Weight1) (*w*)	Error (%)	Training population size			

		1,000	2,000	3,000	5,000
ssGBLUP_0.95	0	0.6162	0.6810	0.7168	0.7579
	10	0.6132	0.6794	0.7160	0.7575
	20	0.6118	0.6779	0.7155	0.7573
	30	0.6093	0.6767	0.7144	0.7568
	40	0.6075	0.6760	0.7139	0.7567
ssGBLUP_0.90	0	0.5996	0.6615	0.6960	0.7359
	10	0.5964	0.6595	0.6949	0.7351
	20	0.5949	0.6577	0.6941	0.7347
	30	0.5921	0.6562	0.6927	0.7338
	40	0.5902	0.6555	0.6921	0.7336
ssGBLUP_0.85	0	0.5834	0.6422	0.6753	0.7139
	10	0.5799	0.6399	0.6739	0.7127
	20	0.5783	0.6377	0.6729	0.7123
	30	0.5753	0.6360	0.6713	0.7109
	40	0.5732	0.6353	0.6706	0.7108

EBV, estimated breeding value; ssGBLUP, single-step genomic best linear unbiased prediction.

1) ssGBLUP_0.95, *w* = 0.95; ssGBLUP_0.90, *w* = 0.90; ssGBLUP_0.85, *w* = 0.85.
